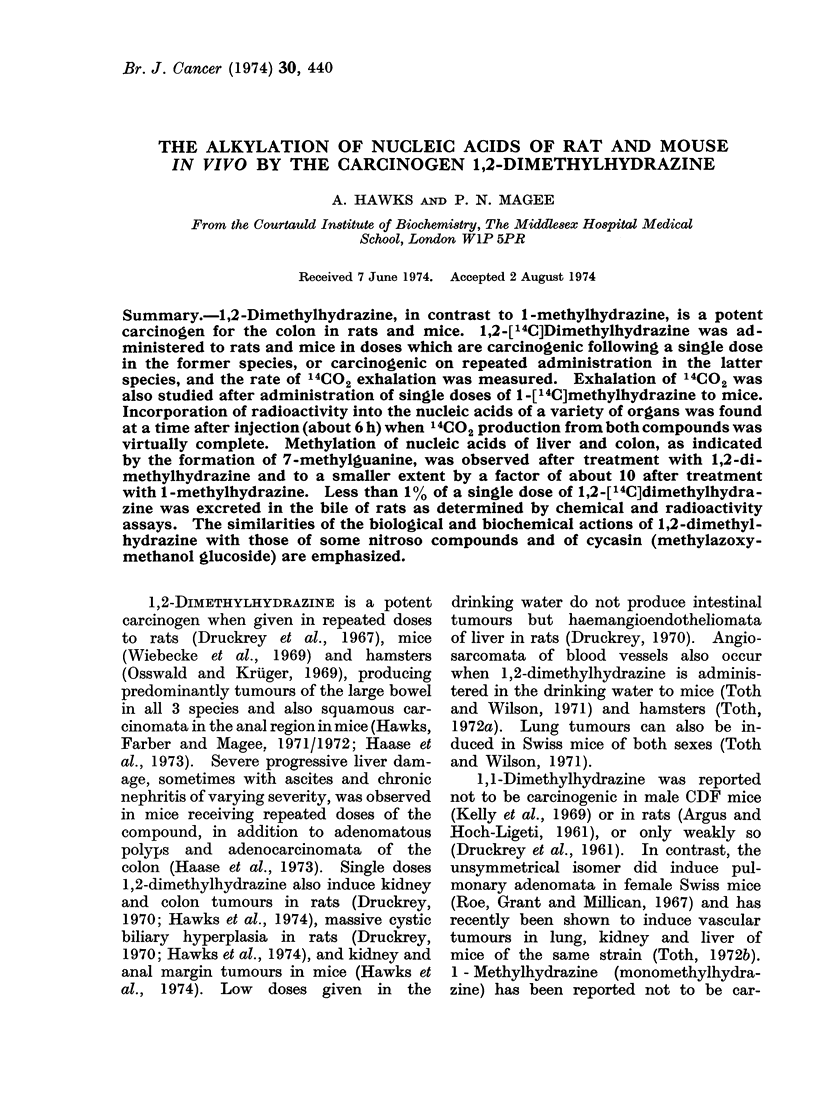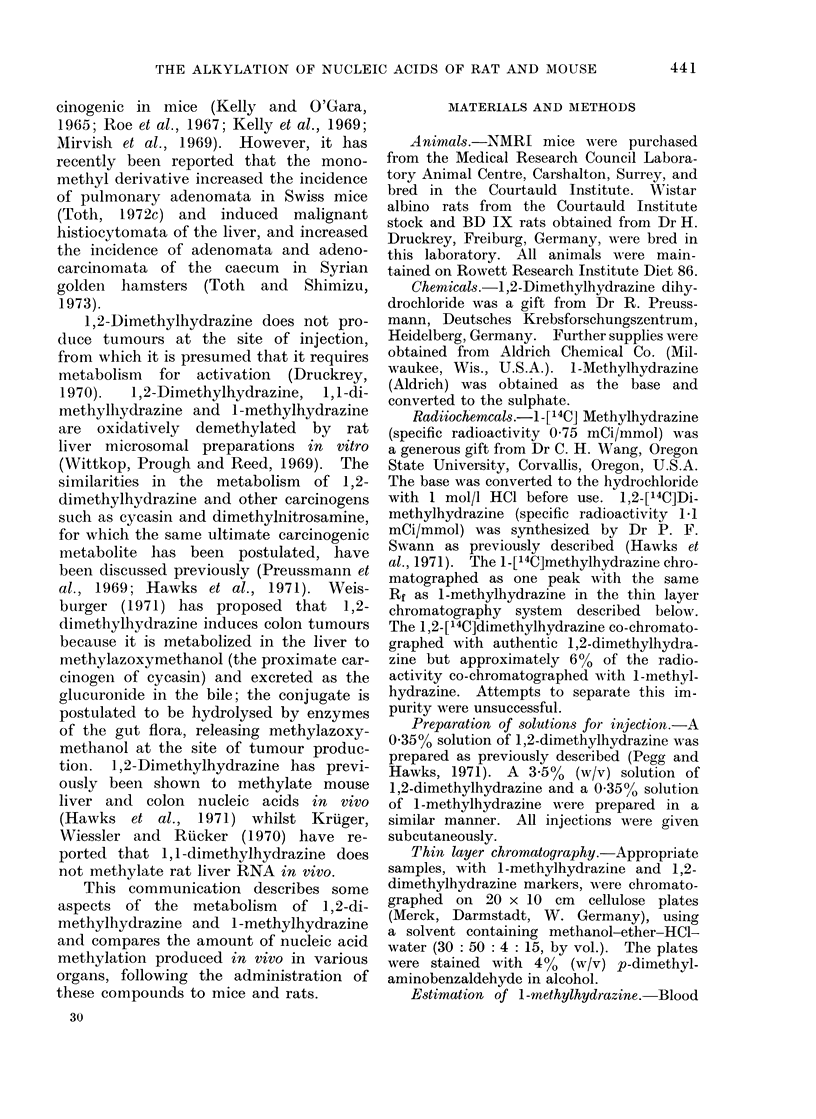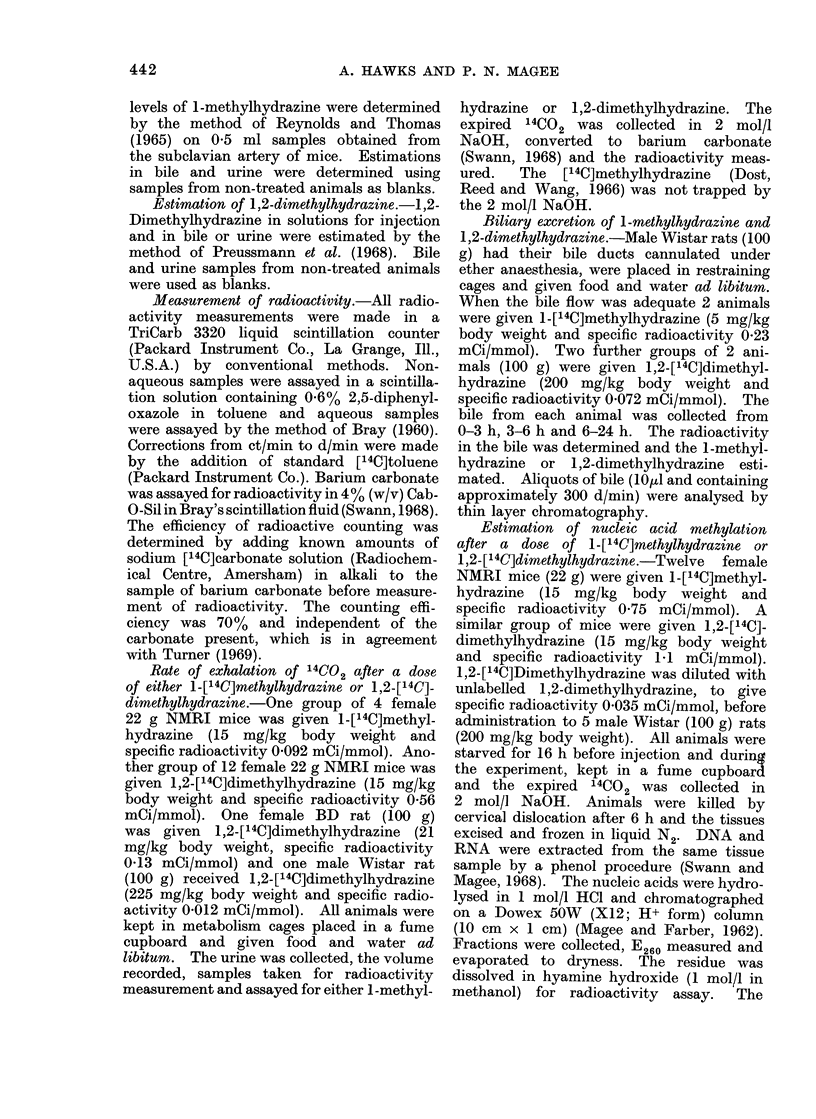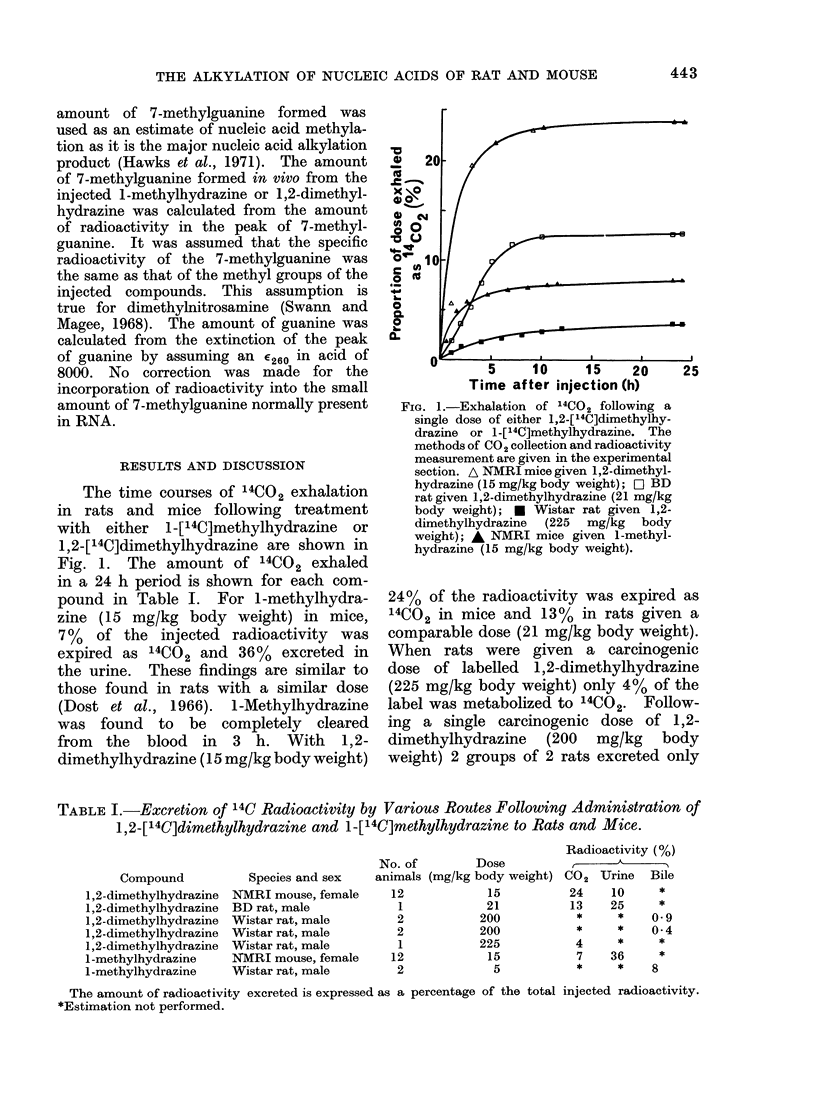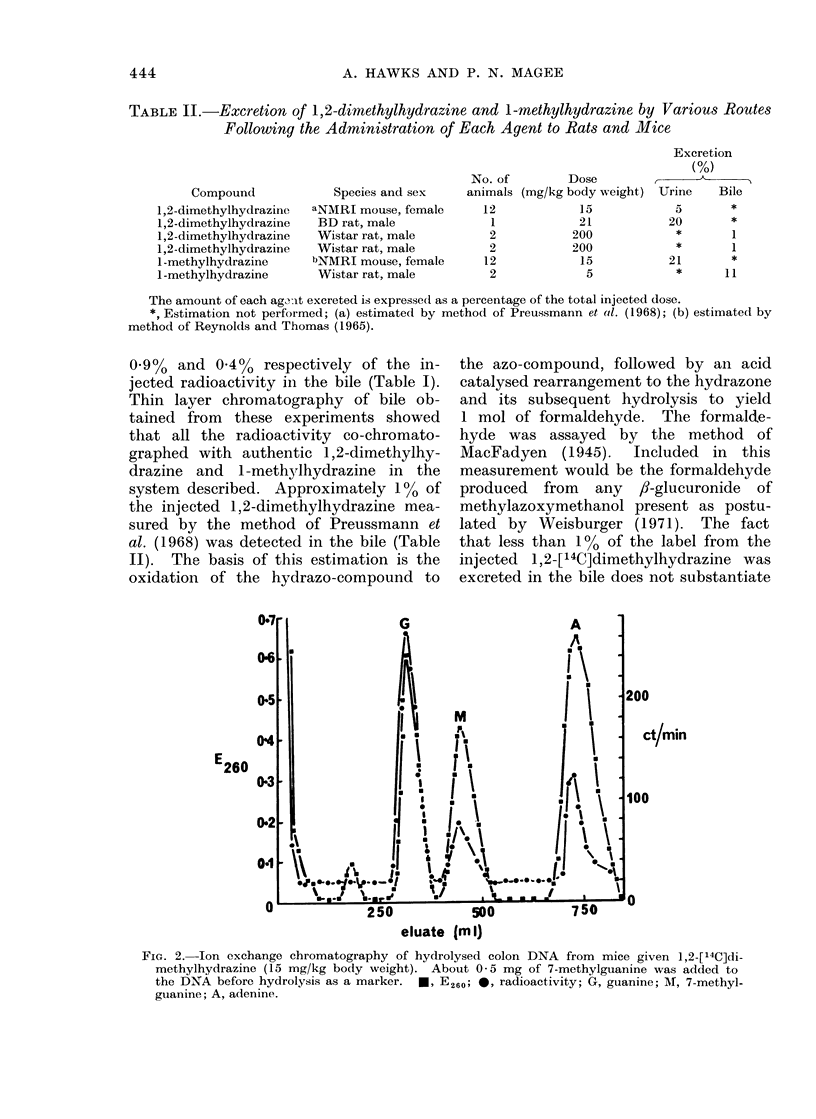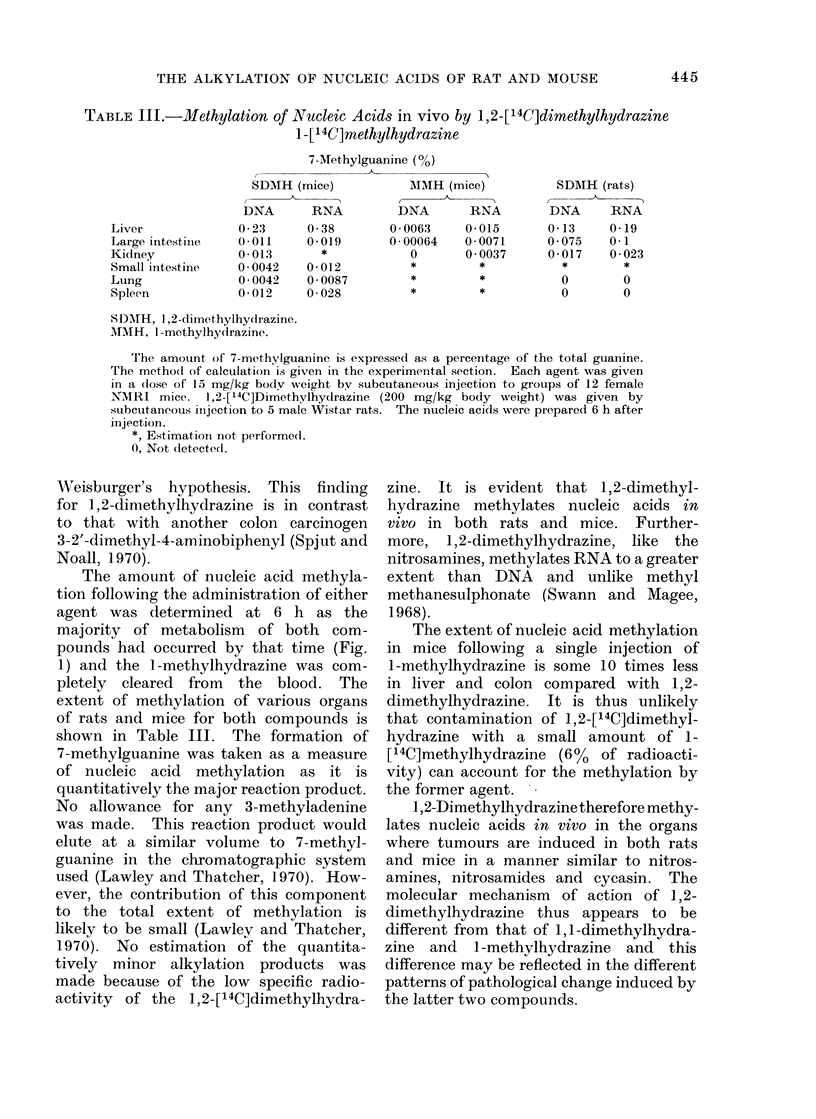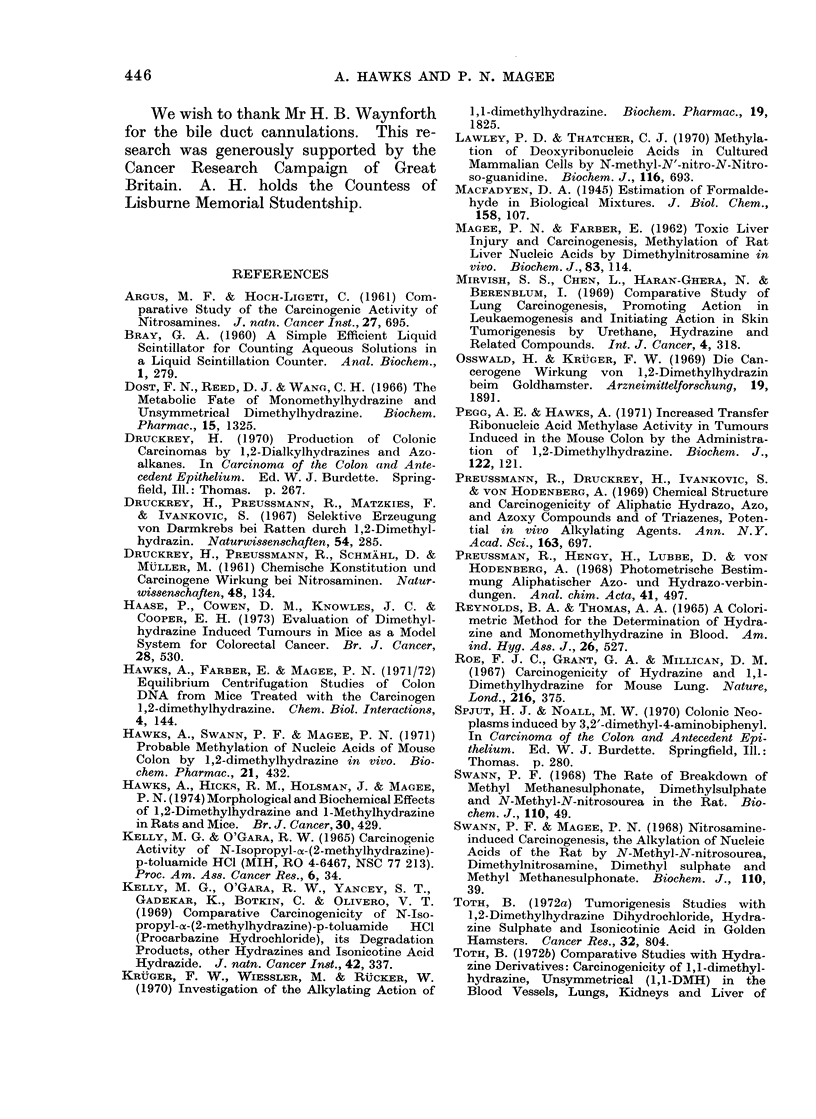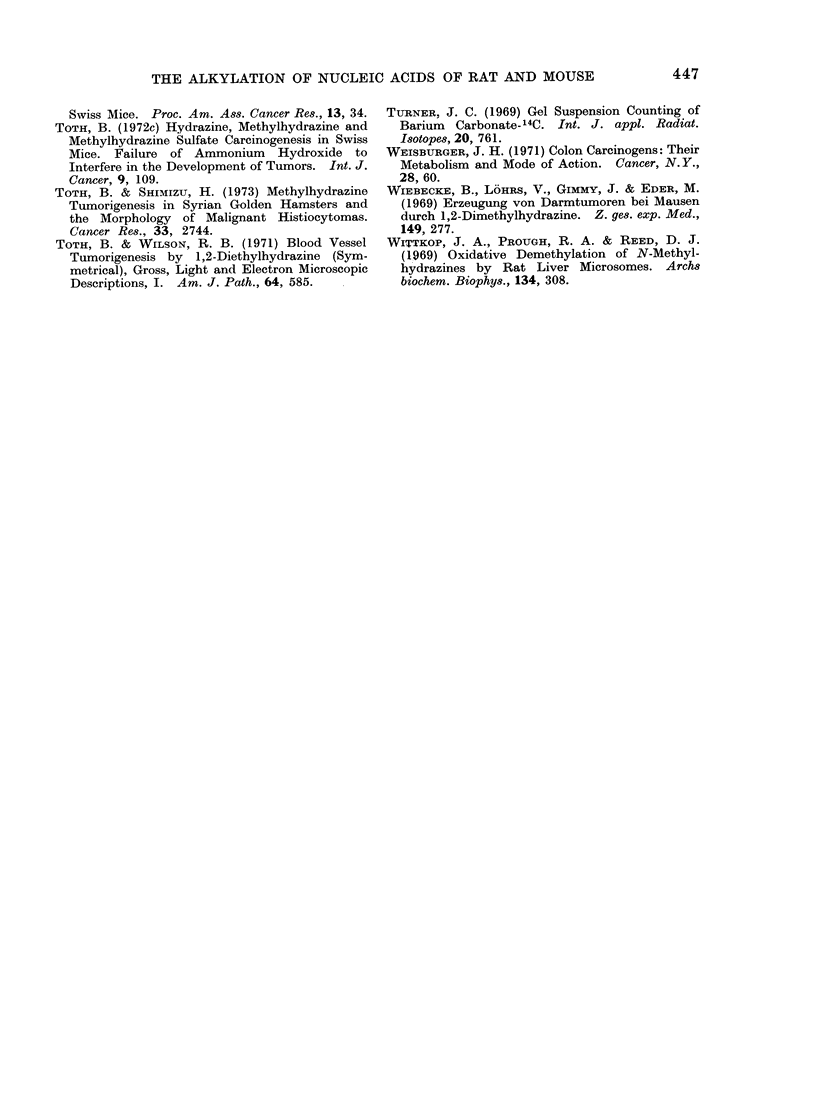# The Alkylation of Nucleic Acids of Rat and Mouse In Vivo by the Carcinogen 1,2-Dimethylhydrazine

**DOI:** 10.1038/bjc.1974.218

**Published:** 1974-11

**Authors:** A. Hawks, P. N. Magee

## Abstract

1,2-Dimethylhydrazine, in contrast to 1-methylhydrazine, is a potent carcinogen for the colon in rats and mice. 1,2-[^14^C]Dimethylhydrazine was administered to rats and mice in doses which are carcinogenic following a single dose in the former species, or carcinogenic on repeated administration in the latter species, and the rate of ^14^CO_2_ exhalation was measured. Exhalation of ^14^CO_2_ was also studied after administration of single doses of 1-[^14^C]methylhydrazine to mice. Incorporation of radioactivity into the nucleic acids of a variety of organs was found at a time after injection (about 6 h) when ^14^CO_2_ production from both compounds was virtually complete. Methylation of nucleic acids of liver and colon, as indicated by the formation of 7-methylguanine, was observed after treatment with 1,2-dimethylhydrazine and to a smaller extent by a factor of about 10 after treatment with 1-methylhydrazine. Less than 1% of a single dose of 1,2-[^14^C]dimethylhydrazine was excreted in the bile of rats as determined by chemical and radioactivity assays. The similarities of the biological and biochemical actions of 1,2-dimethylhydrazine with those of some nitroso compounds and of cycasin (methylazoxymethanol glucoside) are emphasized.


*Br. J. Cancer* (1974) **30**, 440

### THE ALKYLATION OF NUCLEIC ACIDS OF RAT AND MOUSE *IN VIVO* BY THE CARCINOGEN 1,2-DIMETHYLHYDRAZINE

A. HAWKS AND P. N. MAGEE

*From the Courtauld Institute of Biochemistry, The Middlesex Hospital Medical School, London W1P 5PR*

Received 7 June 1974. Accepted 2 August 1974

**Summary.—1,2-Dimethylhydrazine, in contrast to 1-methylhydrazine, is a potent carcinogen for the colon in rats and mice. 1,2-[$^{14}$C]Dimethylhydrazine was administered to rats and mice in doses which are carcinogenic following a single dose in the former species, or carcinogenic on repeated administration in the latter species, and the rate of $^{14}CO_2$ exhalation was measured. Exhalation of $^{14}CO_2$ was also studied after administration of single doses of 1-[$^{14}$C]methylhydrazine to mice. Incorporation of radioactivity into the nucleic acids of a variety of organs was found at a time after injection (about 6 h) when $^{14}CO_2$ production from both compounds was virtually complete. Methylation of nucleic acids of liver and colon, as indicated by the formation of 7-methylguanine, was observed after treatment with 1,2-dimethylhydrazine and to a smaller extent by a factor of about 10 after treatment with 1-methylhydrazine. Less than 1% of a single dose of 1,2-[$^{14}$C]dimethylhydrazine was excreted in the bile of rats as determined by chemical and radioactivity assays. The similarities of the biological and biochemical actions of 1,2-dimethylhydrazine with those of some nitroso compounds and of cycasin (methylazoxymethanol glucoside) are emphasized.**

1,2-DIMETHYLHYDRAZINE is a potent carcinogen when given in repeated doses to rats (Druckrey *et al.*, 1967), mice (Wiebecke *et al.*, 1969) and hamsters (Osswald and Krüger, 1969), producing predominantly tumours of the large bowel in all 3 species and also squamous carcinomata in the anal region in mice (Hawks, Farber and Magee, 1971/1972; Haase *et al.*, 1973). Severe progressive liver damage, sometimes with ascites and chronic nephritis of varying severity, was observed in mice receiving repeated doses of the compound, in addition to adenomatous polyps and adenocarcinomata of the colon (Haase *et al.*, 1973). Single doses 1,2-dimethylhydrazine also induce kidney and colon tumours in rats (Druckrey, 1970; Hawks *et al.*, 1974), massive cystic biliary hyperplasia in rats (Druckrey, 1970; Hawks *et al.*, 1974), and kidney and anal margin tumours in mice (Hawks *et al.*, 1974). Low doses given in the drinking water do not produce intestinal tumours but haemangioendotheliomata of liver in rats (Druckrey, 1970). Angiosarcomata of blood vessels also occur when 1,2-dimethylhydrazine is administered in the drinking water to mice (Toth and Wilson, 1971) and hamsters (Toth, 1972*a*). Lung tumours can also be induced in Swiss mice of both sexes (Toth and Wilson, 1971).

1,1-Dimethylhydrazine was reported not to be carcinogenic in male CDF mice (Kelly *et al.*, 1969) or in rats (Argus and Hoch-Ligeti, 1961), or only weakly so (Druckrey *et al.*, 1961). In contrast, the unsymmetrical isomer did induce pulmonary adenomata in female Swiss mice (Roe, Grant and Millican, 1967) and has recently been shown to induce vascular tumours in lung, kidney and liver of mice of the same strain (Toth, 1972*b*). 1 - Methylhydrazine (monomethylhydrazine) has been reported not to be carcinogenic in mice (Kelly and O'Gara, 1965; Roe *et al.*, 1967; Kelly *et al.*, 1969; Mirvish *et al.*, 1969). However, it has recently been reported that the monomethyl derivative increased the incidence of pulmonary adenomata in Swiss mice (Toth, 1972*c*) and induced malignant histiocytomata of the liver, and increased the incidence of adenomata and adenocarcinomata of the caecum in Syrian golden hamsters (Toth and Shimizu, 1973).

1,2-Dimethylhydrazine does not produce tumours at the site of injection, from which it is presumed that it requires metabolism for activation (Druckrey, 1970). 1,2-Dimethylhydrazine, 1,1-dimethylhydrazine and 1-methylhydrazine are oxidatively demethylated by rat liver microsomal preparations *in vitro* (Wittkop, Prough and Reed, 1969). The similarities in the metabolism of 1,2-dimethylhydrazine and other carcinogens such as cycasin and dimethylnitrosamine, for which the same ultimate carcinogenic metabolite has been postulated, have been discussed previously (Preussmann *et al.*, 1969; Hawks *et al.*, 1971). Weisburger (1971) has proposed that 1,2-dimethylhydrazine induces colon tumours because it is metabolized in the liver to methylazoxymethanol (the proximate carcinogen of cycasin) and excreted as the glucuronide in the bile; the conjugate is postulated to be hydrolysed by enzymes of the gut flora, releasing methylazoxymethanol at the site of tumour production. 1,2-Dimethylhydrazine has previously been shown to methylate mouse liver and colon nucleic acids *in vivo* (Hawks *et al.*, 1971) whilst Krüger, Wiessler and Rücker (1970) have reported that 1,1-dimethylhydrazine does not methylate rat liver RNA *in vivo*.

This communication describes some aspects of the metabolism of 1,2-dimethylhydrazine and 1-methylhydrazine and compares the amount of nucleic acid methylation produced *in vivo* in various organs, following the administration of these compounds to mice and rats.

### MATERIALS AND METHODS

*Animals.*—NMRI mice were purchased from the Medical Research Council Laboratory Animal Centre, Carshalton, Surrey, and bred in the Courtauld Institute. Wistar albino rats from the Courtauld Institute stock and BD IX rats obtained from Dr H. Druckrey, Freiburg, Germany, were bred in this laboratory. All animals were maintained on Rowett Research Institute Diet 86.

*Chemicals.*—1,2-Dimethylhydrazine dihydrochloride was a gift from Dr R. Preussmann, Deutsches Krebsforschungszentrum, Heidelberg, Germany. Further supplies were obtained from Aldrich Chemical Co. (Milwaukee, Wis., U.S.A.). 1-Methylhydrazine (Aldrich) was obtained as the base and converted to the sulphate.

*Radiiochemcals.*—1-[$^{14}$C] Methylhydrazine (specific radioactivity 0·75 mCi/mmol) was a generous gift from Dr C. H. Wang, Oregon State University, Corvallis, Oregon, U.S.A. The base was converted to the hydrochloride with 1 mol/l HCl before use. 1,2-[$^{14}$C]Dimethylhydrazine (specific radioactivity 1·1 mCi/mmol) was synthesized by Dr P. F. Swann as previously described (Hawks *et al.*, 1971). The 1-[$^{14}$C]methylhydrazine chromatographed as one peak with the same $R_f$ as 1-methylhydrazine in the thin layer chromatography system described below. The 1,2-[$^{14}$C]dimethylhydrazine co-chromatographed with authentic 1,2-dimethylhydrazine but approximately 6% of the radioactivity co-chromatographed with 1-methylhydrazine. Attempts to separate this impurity were unsuccessful.

*Preparation of solutions for injection.*—A 0·35% solution of 1,2-dimethylhydrazine was prepared as previously described (Pegg and Hawks, 1971). A 3·5% (w/v) solution of 1,2-dimethylhydrazine and a 0·35% solution of 1-methylhydrazine were prepared in a similar manner. All injections were given subcutaneously.

*Thin layer chromatography.*—Appropriate samples, with 1-methylhydrazine and 1,2-dimethylhydrazine markers, were chromatographed on 20 × 10 cm cellulose plates (Merck, Darmstadt, W. Germany), using a solvent containing methanol–ether–HCl–water (30 : 50 : 4 : 15, by vol.). The plates were stained with 4% (w/v) *p*-dimethylaminobenzaldehyde in alcohol.

*Estimation of 1-methylhydrazine.*—Blood levels of 1-methylhydrazine were determined by the method of Reynolds and Thomas (1965) on 0·5 ml samples obtained from the subclavian artery of mice. Estimations in bile and urine were determined using samples from non-treated animals as blanks.

*Estimation of 1,2-dimethylhydrazine*.—1,2-Dimethylhydrazine in solutions for injection and in bile or urine were estimated by the method of Preussmann *et al.* (1968). Bile and urine samples from non-treated animals were used as blanks.

*Measurement of radioactivity*.—All radioactivity measurements were made in a TriCarb 3320 liquid scintillation counter (Packard Instrument Co., La Grange, Ill., U.S.A.) by conventional methods. Non-aqueous samples were assayed in a scintillation solution containing 0·6% 2,5-diphenyloxazole in toluene and aqueous samples were assayed by the method of Bray (1960). Corrections from ct/min to d/min were made by the addition of standard [$^{14}$C]toluene (Packard Instrument Co.). Barium carbonate was assayed for radioactivity in 4% (w/v) Cab-O-Sil in Bray's scintillation fluid (Swann, 1968). The efficiency of radioactive counting was determined by adding known amounts of sodium [$^{14}$C]carbonate solution (Radiochemical Centre, Amersham) in alkali to the sample of barium carbonate before measurement of radioactivity. The counting efficiency was 70% and independent of the carbonate present, which is in agreement with Turner (1969).

*Rate of exhalation of $^{14}CO_2$ after a dose of either 1-[$^{14}$C]methylhydrazine or 1,2-[$^{14}$C]-dimethylhydrazine*.—One group of 4 female 22 g NMRI mice was given 1-[$^{14}$C]methylhydrazine (15 mg/kg body weight and specific radioactivity 0·092 mCi/mmol). Another group of 12 female 22 g NMRI mice was given 1,2-[$^{14}$C]dimethylhydrazine (15 mg/kg body weight and specific radioactivity 0·56 mCi/mmol). One female BD rat (100 g) was given 1,2-[$^{14}$C]dimethylhydrazine (21 mg/kg body weight, specific radioactivity 0·13 mCi/mmol) and one male Wistar rat (100 g) received 1,2-[$^{14}$C]dimethylhydrazine (225 mg/kg body weight and specific radioactivity 0·012 mCi/mmol). All animals were kept in metabolism cages placed in a fume cupboard and given food and water *ad libitum*. The urine was collected, the volume recorded, samples taken for radioactivity measurement and assayed for either 1-methylhydrazine or 1,2-dimethylhydrazine. The expired $^{14}CO_2$ was collected in 2 mol/l NaOH, converted to barium carbonate (Swann, 1968) and the radioactivity measured. The [$^{14}$C]methylhydrazine (Dost, Reed and Wang, 1966) was not trapped by the 2 mol/l NaOH.

*Biliary excretion of 1-methylhydrazine and 1,2-dimethylhydrazine*.—Male Wistar rats (100 g) had their bile ducts cannulated under ether anaesthesia, were placed in restraining cages and given food and water *ad libitum*. When the bile flow was adequate 2 animals were given 1-[$^{14}$C]methylhydrazine (5 mg/kg body weight and specific radioactivity 0·23 mCi/mmol). Two further groups of 2 animals (100 g) were given 1,2-[$^{14}$C]dimethylhydrazine (200 mg/kg body weight and specific radioactivity 0·072 mCi/mmol). The bile from each animal was collected from 0–3 h, 3–6 h and 6–24 h. The radioactivity in the bile was determined and the 1-methylhydrazine or 1,2-dimethylhydrazine estimated. Aliquots of bile (10$\mu$l and containing approximately 300 d/min) were analysed by thin layer chromatography.

*Estimation of nucleic acid methylation after a dose of 1-[$^{14}$C]methylhydrazine or 1,2-[$^{14}$C]dimethylhydrazine*.—Twelve female NMRI mice (22 g) were given 1-[$^{14}$C]methylhydrazine (15 mg/kg body weight and specific radioactivity 0·75 mCi/mmol). A similar group of mice were given 1,2-[$^{14}$C]-dimethylhydrazine (15 mg/kg body weight and specific radioactivity 1·1 mCi/mmol). 1,2-[$^{14}$C]Dimethylhydrazine was diluted with unlabelled 1,2-dimethylhydrazine, to give specific radioactivity 0·035 mCi/mmol, before administration to 5 male Wistar (100 g) rats (200 mg/kg body weight). All animals were starved for 16 h before injection and during the experiment, kept in a fume cupboard and the expired $^{14}CO_2$ was collected in 2 mol/l NaOH. Animals were killed by cervical dislocation after 6 h and the tissues excised and frozen in liquid $N_2$. DNA and RNA were extracted from the same tissue sample by a phenol procedure (Swann and Magee, 1968). The nucleic acids were hydrolysed in 1 mol/l HCl and chromatographed on a Dowex 50W (X12; $H^+$ form) column (10 cm × 1 cm) (Magee and Farber, 1962). Fractions were collected, $E_{260}$ measured and evaporated to dryness. The residue was dissolved in hyamine hydroxide (1 mol/l in methanol) for radioactivity assay. The amount of 7-methylguanine formed was used as an estimate of nucleic acid methylation as it is the major nucleic acid alkylation product (Hawks *et al.*, 1971). The amount of 7-methylguanine formed *in vivo* from the injected 1-methylhydrazine or 1,2-dimethylhydrazine was calculated from the amount of radioactivity in the peak of 7-methylguanine. It was assumed that the specific radioactivity of the 7-methylguanine was the same as that of the methyl groups of the injected compounds. This assumption is true for dimethylnitrosamine (Swann and Magee, 1968). The amount of guanine was calculated from the extinction of the peak of guanine by assuming an $\epsilon_{260}$ in acid of 8000. No correction was made for the incorporation of radioactivity into the small amount of 7-methylguanine normally present in RNA.

### RESULTS AND DISCUSSION

The time courses of $^{14}CO_2$ exhalation in rats and mice following treatment with either 1-[$^{14}$C]methylhydrazine or 1,2-[$^{14}$C]dimethylhydrazine are shown in Fig. 1. The amount of $^{14}CO_2$ exhaled in a 24 h period is shown for each compound in Table I. For 1-methylhydrazine (15 mg/kg body weight) in mice, 7% of the injected radioactivity was expired as $^{14}CO_2$ and 36% excreted in the urine. These findings are similar to those found in rats with a similar dose (Dost *et al.*, 1966). 1-Methylhydrazine was found to be completely cleared from the blood in 3 h. With 1,2-dimethylhydrazine (15 mg/kg body weight)

FIG. 1.—Exhalation of $^{14}CO_2$ following a single dose of either 1,2-[$^{14}$C]dimethylhydrazine or 1-[$^{14}$C]methylhydrazine. The methods of $CO_2$ collection and radioactivity measurement are given in the experimental section. △ NMRI mice given 1,2-dimethylhydrazine (15 mg/kg body weight); □ BD rat given 1,2-dimethylhydrazine (21 mg/kg body weight); ■ Wistar rat given 1,2-dimethylhydrazine (225 mg/kg body weight); ▲ NMRI mice given 1-methylhydrazine (15 mg/kg body weight).

24% of the radioactivity was expired as $^{14}CO_2$ in mice and 13% in rats given a comparable dose (21 mg/kg body weight). When rats were given a carcinogenic dose of labelled 1,2-dimethylhydrazine (225 mg/kg body weight) only 4% of the label was metabolized to $^{14}CO_2$. Following a single carcinogenic dose of 1,2-dimethylhydrazine (200 mg/kg body weight) 2 groups of 2 rats excreted only

TABLE I.—*Excretion of* $^{14}$*C Radioactivity by Various Routes Following Administration of 1,2-[*$^{14}$*C]dimethylhydrazine and 1-[*$^{14}$*C]methylhydrazine to Rats and Mice.*

| Compound | Species and sex | No. of animals | Dose (mg/kg body weight) | Radioactivity (%) | | |
|---|---|---|---|---|---|---|
| | | | | $CO_2$ | Urine | Bile |
| 1,2-dimethylhydrazine | NMRI mouse, female | 12 | 15 | 24 | 10 | * |
| 1,2-dimethylhydrazine | BD rat, male | 1 | 21 | 13 | 25 | * |
| 1,2-dimethylhydrazine | Wistar rat, male | 2 | 200 | * | * | 0·9 |
| 1,2-dimethylhydrazine | Wistar rat, male | 2 | 200 | * | * | 0·4 |
| 1,2-dimethylhydrazine | Wistar rat, male | 1 | 225 | 4 | * | * |
| 1-methylhydrazine | NMRI mouse, female | 12 | 15 | 7 | 36 | * |
| 1-methylhydrazine | Wistar rat, male | 2 | 5 | * | * | 8 |

The amount of radioactivity excreted is expressed as a percentage of the total injected radioactivity.
*Estimation not performed.

TABLE II.—*Excretion of 1,2-dimethylhydrazine and 1-methylhydrazine by Various Routes Following the Administration of Each Agent to Rats and Mice*

| Compound | Species and sex | No. of animals | Dose (mg/kg body weight) | Excretion (%) Urine | Bile |
|---|---|---|---|---|---|
| 1,2-dimethylhydrazine | [a]NMRI mouse, female | 12 | 15 | 5 | * |
| 1,2-dimethylhydrazine | BD rat, male | 1 | 21 | 20 | * |
| 1,2-dimethylhydrazine | Wistar rat, male | 2 | 200 | * | 1 |
| 1,2-dimethylhydrazine | Wistar rat, male | 2 | 200 | * | 1 |
| 1-methylhydrazine | [b]NMRI mouse, female | 12 | 15 | 21 | * |
| 1-methylhydrazine | Wistar rat, male | 2 | 5 | * | 11 |

The amount of each agent excreted is expressed as a percentage of the total injected dose.
*, Estimation not performed; (a) estimated by method of Preussmann *et al.* (1968); (b) estimated by method of Reynolds and Thomas (1965).

0·9% and 0·4% respectively of the injected radioactivity in the bile (Table I). Thin layer chromatography of bile obtained from these experiments showed that all the radioactivity co-chromatographed with authentic 1,2-dimethylhydrazine and 1-methylhydrazine in the system described. Approximately 1% of the injected 1,2-dimethylhydrazine measured by the method of Preussmann *et al.* (1968) was detected in the bile (Table II). The basis of this estimation is the oxidation of the hydrazo-compound to the azo-compound, followed by an acid catalysed rearrangement to the hydrazone and its subsequent hydrolysis to yield 1 mol of formaldehyde. The formaldehyde was assayed by the method of MacFadyen (1945). Included in this measurement would be the formaldehyde produced from any $\beta$-glucuronide of methylazoxymethanol present as postulated by Weisburger (1971). The fact that less than 1% of the label from the injected 1,2-[$^{14}$C]dimethylhydrazine was excreted in the bile does not substantiate

FIG. 2.—Ion exchange chromatography of hydrolysed colon DNA from mice given 1,2-[$^{14}$C]dimethylhydrazine (15 mg/kg body weight). About 0·5 mg of 7-methylguanine was added to the DNA before hydrolysis as a marker. ■, $E_{260}$; ●, radioactivity; G, guanine; M, 7-methylguanine; A, adenine.

TABLE III.—*Methylation of Nucleic Acids* in vivo *by* 1,2-[$^{14}$*C*]*dimethylhydrazine* 1-[$^{14}$*C*]*methylhydrazine*

| | 7-Methylguanine (%) | | | | | |
|---|---|---|---|---|---|---|
| | SDMH (mice) | | MMH (mice) | | SDMH (rats) | |
| | DNA | RNA | DNA | RNA | DNA | RNA |
| Liver | 0·23 | 0·38 | 0·0063 | 0·015 | 0·13 | 0·19 |
| Large intestine | 0·011 | 0·019 | 0·00064 | 0·0071 | 0·075 | 0·1 |
| Kidney | 0·013 | * | 0 | 0·0037 | 0·017 | 0·023 |
| Small intestine | 0·0042 | 0·012 | * | * | * | * |
| Lung | 0·0042 | 0·0087 | * | * | 0 | 0 |
| Spleen | 0·012 | 0·028 | * | * | 0 | 0 |

SDMH, 1,2-dimethylhydrazine.

MMH, 1-methylhydrazine.

The amount of 7-methylguanine is expressed as a percentage of the total guanine. The method of calculation is given in the experimental section. Each agent was given in a dose of 15 mg/kg body weight by subcutaneous injection to groups of 12 female NMRI mice. 1,2-[$^{14}$C]Dimethylhydrazine (200 mg/kg body weight) was given by subcutaneous injection to 5 male Wistar rats. The nucleic acids were prepared 6 h after injection.

*, Estimation not performed.
0, Not detected.

Weisburger's hypothesis. This finding for 1,2-dimethylhydrazine is in contrast to that with another colon carcinogen 3-2′-dimethyl-4-aminobiphenyl (Spjut and Noall, 1970).

The amount of nucleic acid methylation following the administration of either agent was determined at 6 h as the majority of metabolism of both compounds had occurred by that time (Fig. 1) and the 1-methylhydrazine was completely cleared from the blood. The extent of methylation of various organs of rats and mice for both compounds is shown in Table III. The formation of 7-methylguanine was taken as a measure of nucleic acid methylation as it is quantitatively the major reaction product. No allowance for any 3-methyladenine was made. This reaction product would elute at a similar volume to 7-methylguanine in the chromatographic system used (Lawley and Thatcher, 1970). However, the contribution of this component to the total extent of methylation is likely to be small (Lawley and Thatcher, 1970). No estimation of the quantitatively minor alkylation products was made because of the low specific radioactivity of the 1,2-[$^{14}$C]dimethylhydrazine. It is evident that 1,2-dimethylhydrazine methylates nucleic acids *in vivo* in both rats and mice. Furthermore, 1,2-dimethylhydrazine, like the nitrosamines, methylates RNA to a greater extent than DNA and unlike methyl methanesulphonate (Swann and Magee, 1968).

The extent of nucleic acid methylation in mice following a single injection of 1-methylhydrazine is some 10 times less in liver and colon compared with 1,2-dimethylhydrazine. It is thus unlikely that contamination of 1,2-[$^{14}$C]dimethylhydrazine with a small amount of 1-[$^{14}$C]methylhydrazine (6% of radioactivity) can account for the methylation by the former agent.

1,2-Dimethylhydrazine therefore methylates nucleic acids *in vivo* in the organs where tumours are induced in both rats and mice in a manner similar to nitrosamines, nitrosamides and cycasin. The molecular mechanism of action of 1,2-dimethylhydrazine thus appears to be different from that of 1,1-dimethylhydrazine and 1-methylhydrazine and this difference may be reflected in the different patterns of pathological change induced by the latter two compounds.

We wish to thank Mr H. B. Waynforth for the bile duct cannulations. This research was generously supported by the Cancer Research Campaign of Great Britain. A. H. holds the Countess of Lisburne Memorial Studentship.

### REFERENCES

Argus, M. F. & Hoch-Ligeti, C. (1961) Comparative Study of the Carcinogenic Activity of Nitrosamines. *J. natn. Cancer Inst.*, **27**, 695.

Bray, G. A. (1960) A Simple Efficient Liquid Scintillator for Counting Aqueous Solutions in a Liquid Scintillation Counter. *Anal. Biochem.*, **1**, 279.

Dost, F. N., Reed, D. J. & Wang, C. H. (1966) The Metabolic Fate of Monomethylhydrazine and Unsymmetrical Dimethylhydrazine. *Biochem. Pharmac.*, **15**, 1325.

Druckrey, H. (1970) Production of Colonic Carcinomas by 1,2-Dialkylhydrazines and Azoalkanes. In *Carcinoma of the Colon and Antecedent Epithelium.* Ed. W. J. Burdette. Springfield, Ill.: Thomas. p. 267.

Druckrey, H., Preussmann, R., Matzkies, F. & Ivankovic, S. (1967) Selektive Erzeugung von Darmkrebs bei Ratten durch 1,2-Dimethylhydrazin. *Naturwissenschaften*, **54**, 285.

Druckrey, H., Preussmann, R., Schmähl, D. & Müller, M. (1961) Chemische Konstitution und Carcinogene Wirkung bei Nitrosaminen. *Naturwissenschaften*, **48**, 134.

Haase, P., Cowen, D. M., Knowles, J. C. & Cooper, E. H. (1973) Evaluation of Dimethylhydrazine Induced Tumours in Mice as a Model System for Colorectal Cancer. *Br. J. Cancer*, **28**, 530.

Hawks, A., Farber, E. & Magee, P. N. (1971/72) Equilibrium Centrifugation Studies of Colon DNA from Mice Treated with the Carcinogen 1,2-dimethylhydrazine. *Chem. Biol. Interactions*, **4**, 144.

Hawks, A., Swann, P. F. & Magee, P. N. (1971) Probable Methylation of Nucleic Acids of Mouse Colon by 1,2-dimethylhydrazine *in vivo*. *Biochem. Pharmac.*, **21**, 432.

Hawks, A., Hicks, R. M., Holsman, J. & Magee, P. N. (1974) Morphological and Biochemical Effects of 1,2-Dimethylhydrazine and 1-Methylhydrazine in Rats and Mice. *Br. J. Cancer*, **30**, 429.

Kelly, M. G. & O'Gara, R. W. (1965) Carcinogenic Activity of N-Isopropyl-α-(2-methylhydrazine)-p-toluamide HCl (MIH, RO 4-6467, NSC 77 213). *Proc. Am. Ass. Cancer Res.*, **6**, 34.

Kelly, M. G., O'Gara, R. W., Yancey, S. T., Gadekar, K., Botkin, C. & Olivero, V. T. (1969) Comparative Carcinogenicity of N-Isopropyl-α-(2-methylhydrazine)-p-toluamide HCl (Procarbazine Hydrochloride), its Degradation Products, other Hydrazines and Isonicotine Acid Hydrazide. *J. natn. Cancer Inst.*, **42**, 337.

Krüger, F. W., Wiessler, M. & Rücker, W. (1970) Investigation of the Alkylating Action of 1,1-dimethylhydrazine. *Biochem. Pharmac.*, **19**, 1825.

Lawley, P. D. & Thatcher, C. J. (1970) Methylation of Deoxyribonucleic Acids in Cultured Mammalian Cells by N-methyl-*N*′-nitro-*N*-Nitroso-guanidine. *Biochem. J.*, **116**, 693.

Macfadyen, D. A. (1945) Estimation of Formaldehyde in Biological Mixtures. *J. Biol. Chem.*, **158**, 107.

Magee, P. N. & Farber, E. (1962) Toxic Liver Injury and Carcinogenesis, Methylation of Rat Liver Nucleic Acids by Dimethylnitrosamine *in vivo*. *Biochem. J.*, **83**, 114.

Mirvish, S. S., Chen, L., Haran-Ghera, N. & Berenblum, I. (1969) Comparative Study of Lung Carcinogenesis, Promoting Action in Leukaemogenesis and Initiating Action in Skin Tumorigenesis by Urethane, Hydrazine and Related Compounds. *Int. J. Cancer*, **4**, 318.

Osswald, H. & Krüger, F. W. (1969) Die Cancerogene Wirkung von 1,2-Dimethylhydrazin beim Goldhamster. *Arzneimittelforschung*, **19**, 1891.

Pegg, A. E. & Hawks, A. (1971) Increased Transfer Ribonucleic Acid Methylase Activity in Tumours Induced in the Mouse Colon by the Administration of 1,2-Dimethylhydrazine. *Biochem. J.*, **122**, 121.

Preussmann, R., Druckrey, H., Ivankovic, S. & von Hodenberg, A. (1969) Chemical Structure and Carcinogenicity of Aliphatic Hydrazo, Azo, and Azoxy Compounds and of Triazenes, Potential *in vivo* Alkylating Agents. *Ann. N.Y. Acad. Sci.*, **163**, 697.

Preussmann, R., Hengy, H., Lubbe, D. & von Hodenberg, A. (1968) Photometrische Bestimmung Aliphatischer Azo- und Hydrazo-verbindungen. *Anal. chim. Acta*, **41**, 497.

Reynolds, B. A. & Thomas, A. A. (1965) A Colorimetric Method for the Determination of Hydrazine and Monomethylhydrazine in Blood. *Am. ind. Hyg. Ass. J.*, **26**, 527.

Roe, F. J. C., Grant, G. A. & Millican, D. M. (1967) Carcinogenicity of Hydrazine and 1,1-Dimethylhydrazine for Mouse Lung. *Nature, Lond.*, **216**, 375.

Spjut, H. J. & Noall, M. W. (1970) Colonic Neoplasms induced by 3,2′-dimethyl-4-aminobiphenyl. In *Carcinoma of the Colon and Antecedent Epithelium.* Ed. W. J. Burdette. Springfield, Ill.: Thomas. p. 280.

Swann, P. F. (1968) The Rate of Breakdown of Methyl Methanesulphonate, Dimethylsulphate and *N*-Methyl-*N*-nitrosourea in the Rat. *Biochem. J.*, **110**, 49.

Swann, P. F. & Magee, P. N. (1968) Nitrosamine-induced Carcinogenesis, the Alkylation of Nucleic Acids of the Rat by *N*-Methyl-*N*-nitrosourea, Dimethylnitrosamine, Dimethyl sulphate and Methyl Methanesulphonate. *Biochem. J.*, **110**, 39.

Toth, B. (1972*a*) Tumorigenesis Studies with 1,2-Dimethylhydrazine Dihydrochloride, Hydrazine Sulphate and Isonicotinic Acid in Golden Hamsters. *Cancer Res.*, **32**, 804.

Toth, B. (1972*b*) Comparative Studies with Hydrazine Derivatives: Carcinogenicity of 1,1-dimethylhydrazine, Unsymmetrical (1,1-DMH) in the Blood Vessels, Lungs, Kidneys and Liver of

Swiss Mice. *Proc. Am. Ass. Cancer Res.*, **13**, 34.

TOTH, B. (1972*c*) Hydrazine, Methylhydrazine and Methylhydrazine Sulfate Carcinogenesis in Swiss Mice. Failure of Ammonium Hydroxide to Interfere in the Development of Tumors. *Int. J. Cancer*, **9**, 109.

TOTH, B. & SHIMIZU, H. (1973) Methylhydrazine Tumorigenesis in Syrian Golden Hamsters and the Morphology of Malignant Histiocytomas. *Cancer Res.*, **33**, 2744.

TOTH, B. & WILSON, R. B. (1971) Blood Vessel Tumorigenesis by 1,2-Diethylhydrazine (Symmetrical), Gross, Light and Electron Microscopic Descriptions, I. *Am. J. Path.*, **64**, 585.

TURNER, J. C. (1969) Gel Suspension Counting of Barium Carbonate-$^{14}$C. *Int. J. appl. Radiat. Isotopes*, **20**, 761.

WEISBURGER, J. H. (1971) Colon Carcinogens: Their Metabolism and Mode of Action. *Cancer, N.Y.*, **28**, 60.

WIEBECKE, B., LÖHRS, V., GIMMY, J. & EDER, M. (1969) Erzeugung von Darmtumoren bei Mausen durch 1,2-Dimethylhydrazine. *Z. ges. exp. Med.*, **149**, 277.

WITTKOP, J. A., PROUGH, R. A. & REED, D. J. (1969) Oxidative Demethylation of *N*-Methylhydrazines by Rat Liver Microsomes. *Archs biochem. Biophys.*, **134**, 308.